# Effects of Dietary Energy Level on Performance, Plasma Parameters, and Central AMPK Levels in Stressed Broilers

**DOI:** 10.3389/fvets.2021.681858

**Published:** 2021-05-28

**Authors:** Xiyi Hu, Xianlei Li, Chuanpi Xiao, Linglian Kong, Qidong Zhu, Zhigang Song

**Affiliations:** ^1^Department of Animal Science, Shandong Agricultural University, Taian, China; ^2^Precision Livestock and Nutrition Unit, Gembloux Agro-Bio Tech, University of Liège, Gembloux, Belgium

**Keywords:** broiler, diet energy level, stress, appetite, AMPK

## Abstract

This study aimed to characterize the effects of diets with different energy levels on the growth performance, plasma parameters, and central AMPK signaling pathway in broilers under dexamethasone (DEX)-induced stress. A total of 216 1-day-old male broiler chickens were allocated to groups fed with high (HED), National Research Council-recommended (control), or low (LED) energy diets. At 10 days old, chickens were treated with or without dexamethasone (DEX, 2 mg/kg body weight) for 3 consecutive days. HED increased broiler average daily gain (ADG) at 10 days old, compared with the LED (*P* < 0.05), while average daily feed intake (ADFI) and feed conversion rate (FCR) decreased as the dietary energy level increased (*P* < 0.05). Chickens fed a HED had higher total protein (TP) content, albumin (ALB), glucose (GLU), total cholesterol (TCHO), high-density lipoprotein (HDL) cholesterol, and low-density lipoprotein (LDL) cholesterol, compared with the control group (*P* < 0.05). At 13 days old, DEX decreased ADG and increased FCR in broilers fed with different energy diets (*P* < 0.05). The DEX-HED group had a higher ADFI than non-DEX treated HED group chickens. In addition, TP, ALB, triglycerides (TG), TCHO, HDL, and LDL content levels in the DEX group were higher than those in the control group (*P* < 0.05). The uric acid (UA) content of the LED group was higher than that of the HED group (*P* < 0.05). Further, gene expression levels of liver kinase B1, AMP-activated protein kinase α1, neuropeptide Y, and GC receptor in the hypothalamus were increased in chickens treated with DEX (*P* < 0.05). There was a trend toward interaction between plasma TCHO and hypothalamic LKB1 expression (0.05 < *P* < 0.1). In conclusion, this study suggests that HED improves growth performance, plasma glucose and total cholesterol at 10 days old broilers, but had no significant effect on performance, plasma parameters, and central AMPK in stressed broilers.

## Introduction

Broiler chickens in intensive poultry production systems are challenged by various stress factors (1), including high temperature, high stock density, and diseases. Those factors may impair productive performance and survival, thereby resulting in financial losses for farmers (2, 3). Under stressful conditions, the total energy budget of an animal must be divided optimally among different physiological functions, such as thermoregulation, growth, and reproduction (4). Stress activates the hypothalamic-pituitary-adrenal (HPA) axis, promoting the release of glucocorticoids (GCs) to induce a physiological response to the stressor (5). GC, which are end products of the HPA axis, regulate the basal and stress-related homeostasis (6, 7) and stimulate the appetite in the central nervous system (CNS), facilitating nutrient uptake (8).

Metabolizable energy (ME), a macronutrient composition of diet, is a pivotal factor that influences the feed efficiency, growth performance, and carcass composition of poultry (9, 10). Many recent studies have addressed human and animal food preferences under stress conditions (11–13). Stress increases the preference for high-fat foods in rodents (14, 15) and in humans (16). Further, some studies have found that high-fat diets affect stress response modulation by reducing the autonomic and HPA axis responses to repeated stressors in rodents (14, 17–19). Under stress, chickens may be able to detect metabolic changes (20) and prefer to consume a high-energy diet (1).

The hypothalamus can integrate signals from the brain, peripheral circulation, and gastrointestinal tract to regulate feeding and energy balance (21, 22). The hypothalamus, particularly the arcuate nucleus (ARC), contains two neuron populations that control feed intake, energy balance, and glucose (GLU) homeostasis, and agouti-related peptide/neuropeptide Y (AgRP/NPY)-releasing neurons are orexigenic in the ARC of avian species and mammals (23–25). AMP-activated protein kinase (AMPK) is involved in the regulation of cellular energy homeostasis, which is activated by an increased AMP to ATP ratio in mammals (26). In addition, the liver kinase B1 (LKB1)/AMPK pathway has a similar function in mammals and chickens (27). GC, via the hypothalamic AMPK pathway, induce a preference for food rich in fat (28).

To date, the effects of different energy diets on stress and its underlying mechanisms in broiler chickens remain unknown. In this study, we conducted experiments using different energy diets, and administered subcutaneous injections of dexamethasone (DEX), a synthetic glucocorticoid, to mimic stress (29–31). The aim of this study was to investigate the effects of different energy level diets on the performance, plasma composition, and central AMPK signaling in stressed broiler chickens.

## Materials and Methods

### Experimental Animals, Design, and Management

A total of 216 1-day-old Arbor Acres male broiler chicks (*Gallus gallus domesticus* L.) were obtained from a local hatchery (Da Bao Hatchery, Tai'an, China) and housed in cages in an environmentally controlled room. The brooding temperature was maintained at 35°C for the first 2 days and then gradually decreased by 2–3°C per week, according to the age of broilers (32, 33). The light regime was composed of 23 h of light and 1 h of darkness, and the ambient humidity was 40–50%. The composition and nutrient levels of the chicken diets used in the experiment are listed in Table 1. All birds received feed and water *ad libitum* during the rearing period. This study was approved by the Shandong Agricultural University and carried out in accordance with the Guidelines for Experimental Animals of the Ministry of Science and Technology (Beijing, China).

**Table 1:** Composition and nutrient levels of experimental diets (dry basis).

**Item**	**Content**		
	**Low-energy diet**	**Normal-energy diet**	**High-energy diet**
**Ingredients**			
Corn	52.38	45.07	37.87
Soybean meal	40.45	41.70	42.88
Soybean oil	2.83	8.85	14.86
Limestone	1.16	1.12	1.08
CaHPO_4_	1.93	1.97	2.01
Choline chloride	0.25	0.25	0.25
NaCl	0.30	0.30	0.30
DL-Met	0.21	0.23	0.24
Mineral premix^a^	0.20	0.20	0.20
Vitamin premix^a^	0.30	0.30	0.30
L-Lys·H_2_SO_4_	0	0.01	0.01
L-Thr	0	0	0
Total	100.00	100.00	100.00
**Nutrient levels**^**b**^			
ME/(MJ/kg)	2.90	3.20	3.50
CP	23.00	23.00	23.00
Ca	1.00	1.00	1.00
NPP	0.45	0.45	0.45
Lys	1.20	1.23	1.24
Met	0.54	0.56	0.56
Met+Cys	0.90	0.91	0.91
Thr	0.86	0.86	0.87
Trp	0.30	0.30	0.30

a *Vitamin and mineral premixes provided the following per kilogram of diet: vitamin A, 9,000 IU; vitamin D_3_, 2,000 IU; vitamin E, 11.0 IU; vitamin K, 1.00 mg; thiamine, 1.20 mg; riboflavin, 5.80 mg; niacin, 66.0 mg; pantothenic acid, 10.0 mg; pyridoxine, 2.60 mg; biotin, 0.20 mg; folic acid, 0.70 mg; vitamin B_12_, 0.012 mg; Mn, 100 mg; Zn, 75.0 mg; Fe, 80.0 mg; I, 0.65 mg; Cu, 8.00 mg; Se, 0.35 mg*.

b *Nutrient levels were calculated values*.

Broiler chickens were randomly divided into three groups, with 12 replicates per group and six chickens per replicate. Dietary treatment groups were as follows: (i) low energy diet (LED, ME = 2,900 kcal/kg), (ii) normal energy diet (control, ME = 3,200 kcal/kg), (iii) high energy diet (HED, ME = 3,500 kcal/kg). At 10 days of age, six cages of chickens in each dietary treatment were randomly assigned to receive subcutaneous injections of DEX (2 mg/kg BM/day for 3 days) or sham injection with saline.

### Sample Collection and Procedures

At 10 days of age, one chick was selected from each cage for blood sample collection. At the end of the experiment (13 days of age), two chickens from each replicate were selected and sacrificed. Blood samples were obtained from the wing vein of each chicken using a heparinized syringe. Plasma was obtained after centrifugation at 400 g (4°C, 10 min) and stored at −20°C for further analysis (34, 35). After obtaining blood samples, the chickens were slaughtered through the intravenous injection of pentobarbital sodium (30 mg/kg body weight) and jugular exsanguination. The hypothalamus was collected, in accordance with the method described by Liu et al. (36), via a 4–5 mm deep incision, made parallel to the base of the brain (37). Hypothalamus samples were flash-frozen in liquid nitrogen and stored at −80°C.

### Growth Performance

The initial body weights of broilers were similar among the three groups (42.63 g in control group, 42.62 g in LED group and 42.52 g in HED group). At 10 and 13 days, broilers in each cage were weighed after 8 h of fasting, feed intake recorded, and replicate recorded values used to calculate the average daily gain (ADG), average daily feed intake (ADFI), and feed conversion rate (FCR) per group.

### Plasma Parameters

Plasma concentrations of various factors were measured spectrophotometrically using commercial diagnostic kits (Jiancheng Bioengineering Institute, Nanjing, P. R. China) as follows: total protein (TP; no. A045-2-2), albumin (ALB; no. A028-1-1), glucose (GLU; no. F006), uric acid (UA; no. C012-1-1), total cholesterol (TCHO; no. A111), triglyceride (TG; no. A110), high-density lipoprotein (HDL) cholesterol (no. A112), and low-density lipoprotein (LDL) cholesterol (no. A113). Plasma TP content was determined by Bradford method. Plasma ALB was measured using colorimetric method. Plasma glucose content was measured using the glucose oxidase method. Plasma UA content was determined using the colorimetric method. Plasma TCHO content was measured using the COD-PAP method. Plasma TG content was measured by the GPO-PAP enzymatic method. Plasma HDL-C and LDL were determined according to the kit instructions.

### RNA Extraction and Analysis

Gene expression in the hypothalamus was quantified by quantitative real-time PCR using total RNA extracted using the Trizol reagent (Invitrogen, San Diego, CA, USA). RNA integrity was assessed through agarose gel electrophoresis. The RNA was quantified by using a DeNovix spectrophotometer (DS-11; DeNovix Inc., Wilmington, DE, USA). RNA purity was verified by determining the absorbance ratio of 260 to 280 nm (OD260/280 = 1.8–2.0). mRNA was reverse transcribed into cDNA using a commercial kit (PrimeScript RT Reagent kit, TaKaRa, Dalian, P.R. China). Real-time PCR was carried out using an Applied Biosystems 7500 Real-time PCR System (Applied Biosystems, Foster, CA, USA). Each RT reaction served as a template in 20 μL PCR reaction mixtures containing 0.2 μmol/L of each primer and SYBR green master mix (Takara, Dalian, Liaoning, and PR China). Primer sequences (Table 2) were designed across exon-intron junctions using the Primer 5.0 software. Reaction conditions were as follows: pre-denaturation at 95°C for 30 s, then 40 cycles of denaturation at 95°C for 5 s, and annealing and extension at 60°C for 34 s. A standard curve was plotted to calculate the efficiency of the real-time PCR primers. Relative mRNA expression levels of genes were calculated using the 2^–ΔΔCt^ method and normalized to the value for β-actin expression. Results were verified using glyceraldehyde-3-phosphate dehydrogenase (GAPDH) levels and levels of the control + saline group were used as a calibrator. All processes were performed in accordance with previously described methods (38–40), and each sample was assayed in triplicate.

**Table 2:** Gene-specific primers.

**Gene symbol^a^**	**Accession number**	**Primer sequence (5′ → 3′)**	**Product size (bp)**
*LKB1*	NM_001045833	F: TGAGAGGGATGCTTGAATACGA	158
		R: ACTTGTCCTTTGTTTCTGGGC	
*NPY*	NM_205473	F: CTCTGAGGCACTACATCAACC	142
		R: ACCACATCGAAGGGTCTTCAA	
*AMPKα1*	NM_001039603	F: CGGAGATAAACAGAAGCACGAG	125
		R: CGATTCAGGATCTTCACTGCAAC	
*CCK*	NM_001001741	F: CAGCAGAGCCTGACAGAACC	121
		R: AGAGAACCTCCCAGTGGAACC	
*GR*	NM-001030731	F: AACCTGCTCTGGCTGACTTCTC	121
		R: CCCATCACTTTCGCATCTGTTT	
*β-actin*	NM_205518.1	F: CTGGCACCTAGCACAATGAA	123
		R: CTGCTTGCTGATCCACATCT	
*GAPDH*	NM_204305	F: ACATGGCATCCAAGGAGTGAG	266
		R: GGGGAGACAGAAGGGAACAGA	

a *LKB1, liver kinase B1; NPY, neuropeptide Y; AMPKα1, AMP-activated protein kinase α1; CCK, cholecystokinin; GR, glucocorticoid receptor; GAPDH, glyceraldehyde phosphate dehydrogenase*.

### Statistical Analysis

Differences between groups receiving different dietary treatments were analyzed using one-way ANOVA and the GLM procedure in Statistical Analysis Systems (SAS) software (version 8.02; SAS Institute Inc., Cary, NC). When differences among individual means were found in ANOVA tests (*P* < 0.05), means were compared using the Tukey's test. A two-way ANOVA model was used to analyze the main effects of diet energy, DEX treatment, and their interaction using SAS software. Results are presented as mean values and pooled standard error of the mean (SEM). For statistical analysis of ADG and ADFI, one cage was considered as one replicate. For measurement of plasma parameters and gene expression in the hypothalamus, one chick from each cage was sampled, and one replicate comprised one chick. *P* < 0.05 indicated statistical significance.

## Results

### Effects of Dietary Energy Levels and DEX on Broiler Chickens Growth Performance (Presented the Baseline Situation, Without DEX, and Then With DEX)

The effects of dietary energy levels on broiler performance are summarized in Table 3. From days 0 to 10, chicks fed with the HED had greater ADG and lower ADFI (*P* < 0.05) than those fed with the LED, while LED treatment resulted in higher ADFI and FCR (*P* < 0.05) than the control and HED treatments. The effects of DEX on the performance of broilers fed with different energy diets are presented in Table 4. ADG was decreased in response to DEX injection (*P* = 0.0002). The control group had a higher ADG and lower FCR than the control-DEX group (*P* = 0.0003). Further, ADFI in the LED group was lower than that in the control and HED groups (*P* = 0.0040), irrespective of DEX injection. No significant interaction of diet and DEX treatment on growth performance was detected.

**Table 3:** Effects of dietary energy levels on the performance of 10-day-old broiler chickens.

**Item^2^**	**Dietary treatment**^**1**^			***P*-value**
	**Control**	**LED**	**HED**	
BW (g/bird, 1 day)	42.63 ± 0.11	42.62 ± 0.12	42.52 ± 0.19	0.82
BW (g/bird, 10 days)	263.28 ± 6.51^a^^b^	249.53 ± 6.24^b^	270.63 ± 6.13^a^	0.08
ADG (g/bird/day)	16.97 ± 0.50^a^^b^	15.92 ± 0.48^b^	17.54 ± 0.48^a^	0.08
ADFI (g/bird/day)	17.14 ± 0.2^b^	18.31 ± 0.19^a^	16.84 ± 0.22^b^	0.00
FCR	1.01 ± 0.02^b^	1.15 ± 0.02^a^	0.96 ± 0.007^b^	0.00

1 *LED, low energy diet (2,900 kcal/kg); control, National Research Council-recommended energy diet (3,200 kcal/kg); HED, high energy diet (3,500 kcal/kg)*.

2 *BW, body weight; ADG, average daily gain; ADFI, average daily feed intake; FCR, feed conversion rate*.

*The values are presented as mean ± SEM (N = 12)*.

a,b *Mean values in a row sharing no common superscript are significantly different (P < 0.05)*.

**Table 4:** Effects of DEX on the performance of 13-day-old broilers fed with different energy level diets^1^.

**Item^2^**	**LED**		**Control**		**HED**		**SEM**	***P*****-value**		
	**Saline**	**DEX**	**Saline**	**DEX**	**Saline**	**DEX**		**Diet**	**DEX**	**Diet × DEX**
BW (g/bird, 13 days)	386.43^a^	335.18^b^	408.39^a^	331.07^b^	388.33^a^	312.38^b^	11.80	0.29	0.00	0.44
ADG (g/bird/day)	40.42^a^	19.69^b^	45.66^a^	25.31^b^	40.77^a^	33.07^ab^	4.15	0.23	0.00	0.27
ADFI (g/bird/day)	61.46^b^	62.22^b^	63.84^b^	65.15^ab^	65.17^ab^	68.33^a^	1.21	0.00	0.11	0.62
FCR	1.52^b^	3.16^a^	1.39^b^	2.58^a^	1.60^b^	2.07^a^	0.33	0.43	0.00	0.30

*SEM = pooled standard error of the means*.

1 *LED, low energy diet (2900 kcal/kg); control, National Research Council-recommended energy diet (3,200 kcal/kg); HED, high energy diet (3,500 kcal/kg); DEX, dexamethasone (2 mg/kg body weight)*.

2 *BW, body weight; ADG, average daily gain; ADFI, average daily feed intake; FCR, feed conversion rate*.

*Data represented the mean value of six cages per treatment*.

a,b *Mean values in a row sharing no common superscript are significantly different (P < 0.05)*.

### Effects of Dietary Energy Level and DEX on Broiler Chickens Plasma Parameters (Presented the Baseline Situation, Without DEX, and Then With DEX)

The effects of dietary energy levels on the plasma parameters of broilers are presented in Table 5. HED treatment resulted in higher plasma TP, ALB, GLU, TCHO, HDL, and LDL (*P* < 0.05) relative to the control treatment group. UA concentration was increased in the LED group compared with that in the HED group (*P* < 0.05). No significant differences in plasma parameters were observed between the LED and control treatment groups. TG levels were not influenced by dietary treatment. The effects of DEX on the plasma parameters of broilers fed with different energy level diets are shown in Table 6; various plasma parameters were influenced by DEX injection. The contents of TP, ALB, TG, TCHO, HDL, and LDL in the control-DEX group were higher than those in the control group (*P* < 0.0001, *P* < 0.0001, *P* = 0.0740, *P* < 0.0001, *P* < 0.0001, and *P* < 0.0001, respectively). Further, UA content in both the LED and HED groups decreased in response to DEX (*P* = 0.0101), while the UA content of the LED group was higher than that of the HED group (*P* = 0.0036). In contrast GLU level was influenced by neither dietary energy level nor DEX injection. An interaction of dietary energy level and DEX injection was found to influence TCHO content (*P* = 0.0715), while no there was no evidence of an influence of such an interaction on TP, ALB, UA, GLU, TG, HDL, or LDL content.

**Table 5:** Effects of dietary energy levels on the plasma parameters in 10-day-old broilers.

**Item^2^**	**Dietary treatment**^**1**^			***P*-value**
	**Control**	**LED**	**HED**	
TP, g/L	23.80 ± 0.79^b^	26.19 ± 0.34^ab^	27.53 ± 1.56^a^	0.05
ALB, g/L	7.81 ± 0.41^b^	8.06 ± 0.15^b^	9.12 ± 0.41^a^	0.03
UA, μmol/L	290.13 ± 18.54^ab^	356.25 ± 31.22^a^	250.50 ± 38.05^b^	0.07
GLU, mmol/L	10.52 ± 0.42^b^	10.79 ± 0.32^b^	12.33 ± 0.48^a^	0.01
TG, mmol/L	0.52 ± 0.02	0.51 ± 0.02	0.52 ± 0.04	0.96
TCHO, mmol/L	3.39 ± 0.18^b^	3.93 ± 0.17^ab^	4.28 ± 0.23^a^	0.02
HDL cholesterol, mmol/L	2.76 ± 0.16^b^	3.21 ± 0.14^ab^	3.33 ± 0.21^a^	0.08
LDL cholesterol, mmol/L	0.56 ± 0.03^b^	0.62 ± 0.04^ab^	0.76 ± 0.07^a^	0.03

1 *LED, low-energy diet (2900 kcal/kg); control, National Research Council-recommended energy diet (3,200 kcal/kg); HED, high-energy diet (3,500 kcal/kg)*.

2 *TP, total protein; ALB, albumin; UA, uric acid; GLU, glucose; TG, triglycerides; TCHO, total cholesterol; HDL, high-density lipoprotein; LDL, low-density lipoprotein*.

*The values are presented as mean ± SEM (N = 8)*.

a,b *Mean values in a row sharing no common superscript are significantly different (P < 0.05)*.

**Table 6:** Effects of DEX on plasma parameters in 13-day-old broilers fed with different energy level diets^1^.

**Item^2^**	**LED**		**Control**		**HED**		**SEM**	***P*****-value**		
	**Saline**	**DEX**	**Saline**	**DEX**	**Saline**	**DEX**		**Diet**	**DEX**	**Diet × DEX**
TP, g/L	27.51^b^	35.09^a^	27.17^b^	34.14^a^	26.00^b^	36.83^a^	1.00	0.86	0.00	0.16
ALB, g/L	8.23^b^	10.76^a^	8.19^b^	10.81^a^	8.37^b^	11.58^a^	0.32	0.78	0.00	0.58
UA, μmol/L	324.25^a^	279.60^ab^	301.67^ab^	230.43^bc^	242.00^abc^	180.14^c^	24.22	0.00	0.01	0.88
GLU, mmol/L	11.39	10.82	11.62	10.81	11.74	11.34	0.47	0.56	0.13	0.91
TG, mmol/L	0.69^ab^	0.68^ab^	0.65^ab^	0.81^a^	0.49^b^	0.76^ab^	0.08	0.34	0.07	0.34
TCHO, mmol/L	3.37^c^	4.97^ab^	3.46^c^	4.90^b^	3.18^c^	5.54^a^	0.20	0.87	0.00	0.07
HDL, mmol/L	2.82^b^	4.24^a^	2.87^b^	4.13^a^	3.23^b^	4.82^a^	0.20	0.07	0.00	0.74
LDL, mmol/L	0.56^b^	0.90^a^	0.55^b^	0.87^a^	0.56^b^	0.85^a^	0.04	0.88	0.00	0.82

*SEM = pooled standard error of the means*.

1 *LED, low energy diet (2,900 kcal/kg); control, National Research Council-recommended energy diet (3,200 kcal/kg); HED, high energy diet (3,500 kcal/kg); DEX, dexamethasone (2 mg/kg body weight)*.

2 *TP, total protein; ALB, albumin; UA, uric acid; GLU, glucose; TG, triglycerides; TCHO, total cholesterol; HDL, high-density lipoprotein; LDL, low-density lipoprotein*.

*Data represented the mean value of eight samples per treatment*.

a,b,c *Mean values in a row sharing no common superscript are significantly different (P < 0.05)*.

### Effects of DEX on the Expression Levels of Appetite-Related Genes and AMPK Signaling in Broiler Chickens Fed With Different Energy Level Diet

**Figure 1:**
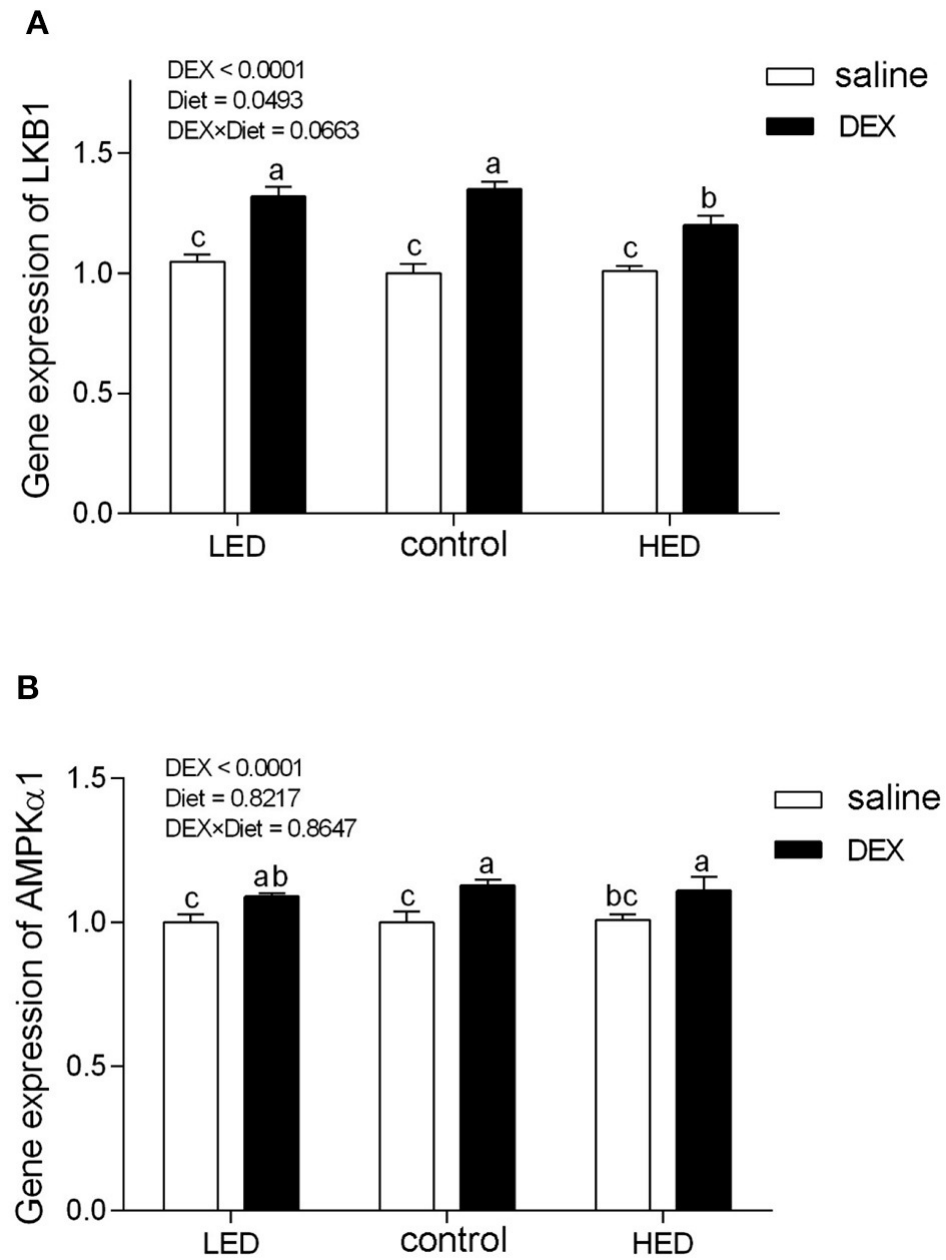
Effects of DEX on the mRNA expression levels of *LKB1* **(A)** and *AMPK*α*1* **(B)** in the hypothalamus of 13-day-old broilers fed with different energy level diets. Values were obtained from duplicates of each sample and are presented as mean ± SEM (*N* = 8); ^a,b,c^Means sharing no common letters differ significantly (*P* < 0.05; ANOVA). LED, low energy diet; control, National Research Council-recommended diet; HED, high energy diet; DEX, dexamethasone.

**Figure 2:**
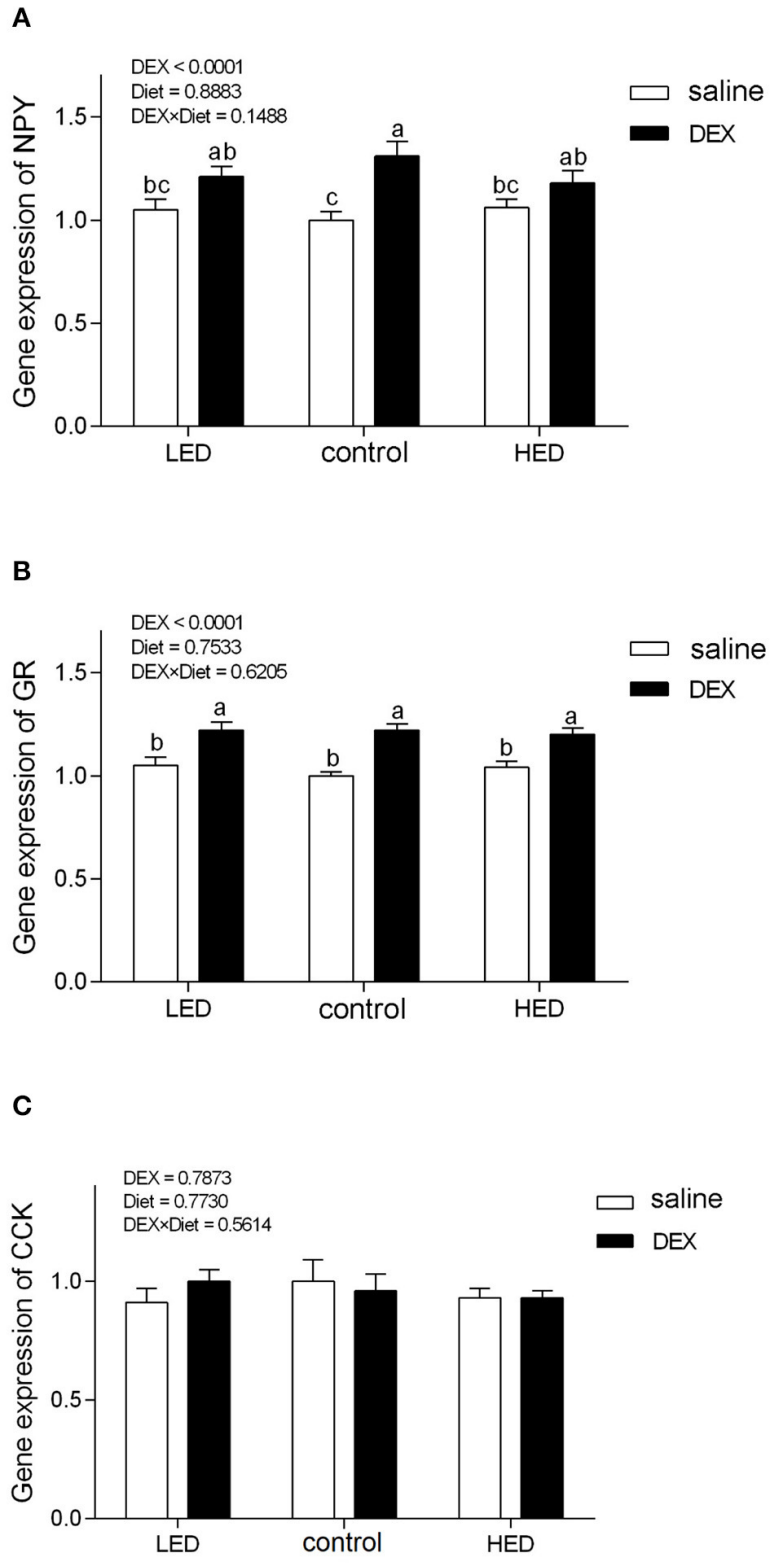
Effects of DEX on the mRNA expression levels of *NPY* **(A)**, *GR* **(B)**, and *CCK* **(C)** in the hypothalamus of 13-day-old broilers fed with different energy level diets. Values were obtained from duplicates of each sample and are presented as mean ± SEM (*N* = 8). ^a,b,c^Means sharing no common letters differ significantly (*P* < 0.05; ANOVA). LED, low energy diet; control, National Research Council-recommended diet; HED, high energy diet; DEX, dexamethasone.

The relative gene expression levels of *AMPK* and appetite-related genes are presented in Figures 1, 2, respectively. DEX treatment increased the gene expression levels of *LKB1, AMPK*α*1, NPY*, and glucocorticoid receptor (*GR*) in hypothalamus tissue from 13-day-old broilers (all *P* < 0.0001). *LKB1* mRNA levels in the LED group were higher than those in the HED group (*P* = 0.0493). In addition, a trend of interaction was observed between DEX and the dietary energy treatments on *LKB1* gene expression in the hypothalamus (*P* = 0.0663). Further, cholecystokinin (*CCK*) mRNA levels in the hypothalamus of 13-day-old broilers were not affected by either DEX or dietary energy level (*P* > 0.1).

## Discussion

### Effects of Dietary Energy Levels and DEX on Broiler Chicken Growth Performance

Dietary energy in broiler nutrition plays a significant and central role in livestock maintenance and production (41). In this study, chicks fed a HED had higher ADG than those fed a LED, consistent with our previous findings (42). The increase in body weight induced by consumption of a HED has been widely verified in human and animal studies (21, 43–45). A LED reduces production performance, due to the low energy intake (46); however, Yuan et al. (1) has found that chicken body weight gain was not altered by dietary energy level. This controversial conclusion may be attributable to the age and the dietary energy level used in the study. Birds consume feed primarily to meet their energy requirements and increase their feed intake in response to dietary energy dilution (41, 47), consistent with our results. Chickens can control their feed intake according to changes in dietary ME concentration (48). The feed intake of broilers fed a low ME diet increased; however, this could not compensate for the poor weight gain and FCR, due to the physical limitations of feed consumption (47). In this study, as dietary energy levels increased, FCR decreased significantly. Several reports have revealed that increased dietary energy can reduce broiler FCR (45, 49, 50). Together, current data suggest that broilers can adjust their feed intake in response to dietary energy levels.

GC can evoke a particular appetite for foods rich in fat or energy (28). DEX had significant effects on ADG and FCR. This observation was consistent with the results of Lv et al. (51), who reported that DEX treatment decreases the body weight gain of broilers as FCR increases. Increased energy expenditure, protein oxidation, and reduced small intestine absorption are responsible for the suppressive effects of GC on growth rate (4, 52, 53). In the present study, the interaction between DEX and dietary energy level had no significant effect on ADG, ADFI, or FCR, similar to the findings of a previous study (4); however, Yuan et al. (1) found that chickens fed a HED had higher BW gain than those receiving a LED on corticosterone treatment, indicating the beneficial effects of a HED. Differences in dietary energy content or fat type may partially account for these discrepancies.

### Effects of Dietary Energy Level and DEX on Broiler Chicken Plasma Parameters

Serum GLU is an important energy source conducive to body tissue growth, whereas the serum ALB and TP reflect protein synthesis functions in the broiler liver, which may be associated with physiological status and growth (54). In birds, UA is the main product of nitrogen metabolism, and its content can reflect the direction of protein metabolism (55). In this study, birds fed with HED showed increased levels of plasma GLU, TP, and ALB at 10 days old. GLU in the blood can be oxidized to provide energy and channeled into pathways for fatty acid synthesis (56). The increased GLU concentration detected in the plasma of HED group chickens suggests a high rate of GLU use in fatty acid synthesis. Increased serum ALB and TP can be related to improved protein digestibility and increased availability of amino acid precursors for protein synthesis (57). In this study, at 13 days old, levels of TP and ALB were affected by treatment with DEX, consistent with the result of Lv et al. (51); however, dietary energy levels did not affect these parameters, in accordance with the findings of Yang et al. (4). The level of UA was affected by diet type and DEX treatment; however, no interaction between diet and DEX was evident. These results indicated that dietary energy level cannot alleviate the influence of stress on protein metabolism.

Lipid metabolism was also assessed in our study. Serum TG, TCHO, HDL-cholesterol, and LDL-cholesterol are key indicators of lipid metabolism balance (58–60). Increased levels of serum TCHO and HDL-cholesterol were observed in the HED group, consistent with the results of previous studies (50, 61, 62). HDL-cholesterol is responsible for the uptake of cholesterol from the peripheral tissues and blood and facilitates its transport back to the liver for catabolism (63), whereas LDL-cholesterol has the opposite function (63). Most fatty acids are synthesized in the liver and transported via LDL for storage as TG in adipose tissue (64). Therefore, in the HED group, cholesterol from the peripheral tissues was transported to the liver, as reported by Ge et al. (50), and TG was deposited in the adipose tissue. In the present experiment, DEX treatment significantly increased the levels of TG, TCHO, HDL-C, and LDL-C, regardless of the dietary energy level, as also revealed in previous studies (1, 51). A trend toward an effect of interaction of dietary energy level and DEX treatment on plasma TCHO levels was detected. This finding is consistent with previous research, revealing that the effect of GC on TG is minimal when the diet composition maintains a low lipid flux, but becomes highly significant when the dietary lipid flux increases (65).

### Effects of DEX on the Expression Levels of Appetite-Related Genes and AMPK Signaling in Broiler Chickens Fed With Different Energy Level Diets

In the CNS, the hypothalamus is vital in coordinating feed intake and regulating energy homeostasis in mammals and birds (66–68), and various orexigenic and anorexigenic neuropeptides have been identified in the hypothalamus of mammals and poultry (5). Hypothalamic NPY is a potently orexigenic neuropeptide, which can increase appetite in mammals and birds (23, 69, 70). Consistent with previous findings in rats (71) and chicks (28), our data demonstrate that GC treatment increased hypothalamic NPY levels, suggesting that appetite was stimulated. GR, which belongs to the steroid/sterol/thyroid/retinoid/orphan nuclear receptor superfamily, mediates the majority of the known actions of GC (72, 73) and regulates food intake and energy expenditure (22). In this study*, GR* mRNA levels were increased on treatment with DEX in both dietary energy level groups, indicating that the biological regulation of GC was strengthened. AMPK has emerged as a key molecular regulator with a pivotal role in cellular energy metabolism (74–76). In the CNS, AMPK participates in fasting, inflammation, stress, and other responses (77–80). Once activated, AMPK switches off anabolic pathways (such as fatty acid, TG, and cholesterol syntheses), in favor of catabolic pathways (such as glycolysis and fatty acid oxidation), to sustain energy homeostasis (75, 81). GC activate hypothalamic AMPK activity (82, 83), and this can lead to appetite stimulation (84). In this study, exogenous subcutaneous GC administration significantly increased hypothalamic *NPY, AMPK*α*1*, and *GR* gene expression, while the expression levels of these genes were not altered by dietary energy level; however, level of *LKB1* were affected by diet and DEX. LKB1, a kinase upstream of AMPK, has the same function in poultry and mammals (27); *LKB1* expression was reduced in DEX-treated chicks fed the HED, indicating that HED can partially reduce activation of AMPK signaling. In other words, high energy diet has the potential to inhibit the activation of central AMPK signaling by stress.

## Conclusion

The results of the present study demonstrate that HED improves growth performance, plasma glucose and total cholesterol at 10 days old broilers. Although there is a trend toward an effect of interaction of stress and diet on plasma TCHO content and hypothalamic *LKB1* expression, in general, high energy diet has little effect on performance, plasma parameters and central AMPK in 10-day old stressed broiler chickens.

## Data Availability Statement

The original contributions presented in the study are included in the article, further inquiries can be directed to the corresponding author.

## Ethics Statement

The animal study was reviewed and approved by Guidelines for Experimental Animals of the Ministry of Science and Technology (Beijing, China).

## Author Contributions

XH and ZS conceived and designed the experiments and wrote the paper. XH and XL performed the experiments and analyzed the data. LK, CX, and QZ provided essential reagents. All authors read and approved the final manuscript.

## Conflict of Interest

The authors declare that the research was conducted in the absence of any commercial or financial relationships that could be construed as a potential conflict of interest.

## Abbreviations

DEX: dexamethasone
HED: high energy diet
LED: low energy diet
ADG: average daily gain
ADFI: average daily feed intake
FCR: feed conversion rate
TP: total protein
ALB: albumin
GLU: glucose
TCHO: total cholesterol
HDL: high-density lipoprotein
LDL: low-density lipoprotein
TG: triglycerides
UA: uric acid
HPA: hypothalamic-pituitary-adrenal
GCs: glucocorticoids
CNS: central nervous system
ME: Metabolizable energy
ARC: arcuate nucleus
AgRP: agouti-related peptide
NPY: neuropeptide Y
AMPK: AMP-activated protein kinase
LKB1: liver kinase B1
GR: glucocorticoid receptor
GAPDH: glyceraldehyde-3-phosphate dehydrogenase
CCK: cholecystokinin.
